# *Mycobacterium tuberculosis* Lipolytic Enzymes as Potential Biomarkers for the Diagnosis of Active Tuberculosis

**DOI:** 10.1371/journal.pone.0025078

**Published:** 2011-09-22

**Authors:** Belinda Brust, Mélanie Lecoufle, Edouard Tuaillon, Luc Dedieu, Stéphane Canaan, Viviane Valverde, Laurent Kremer

**Affiliations:** 1 Laboratoire de Dynamique des Interactions Membranaires Normales et Pathologiques, Universités de Montpellier II et I, CNRS UMR 5235, Montpellier, France; 2 INSERM, DIMNP, Montpellier, France; 3 INSERM U1058, Université de Montpellier I, Département de Bactériologie-Virologie, Institut de Recherche en Biothérapie, Centre Hospitalier Universitaire de Montpellier, Montpellier, France; 4 CNRS UPR 9025, Université Aix-Marseille, Enzymologie Interfaciale et Physiologie de la Lipolyse, Marseille, France; 5 Clinical Microbiology Division, Bio-Rad Laboratories, Steenvoorde, France; University of Maryland, United States of America

## Abstract

**Background:**

New diagnosis tests are urgently needed to address the global tuberculosis (TB) burden and to improve control programs especially in resource-limited settings. An effective *in vitro* diagnostic of TB based on serological methods would be regarded as an attractive progress because immunoassays are simple, rapid, inexpensive, and may offer the possibility to detect cases missed by standard sputum smear microscopy. However, currently available serology tests for TB are highly variable in sensitivity and specificity. Lipolytic enzymes have recently emerged as key factors in lipid metabolization during dormancy and/or exit of the non-replicating growth phase, a prerequisite step of TB reactivation. The focus of this study was to analyze and compare the potential of four *Mycobacterium tuberculosis* lipolytic enzymes (LipY, Rv0183, Rv1984c and Rv3452) as new markers in the serodiagnosis of active TB.

**Methods:**

Recombinant proteins were produced and used in optimized ELISA aimed to detect IgG and IgM serum antibodies against the four lipolytic enzymes. The capacity of the assays to identify infection was evaluated in patients with either active TB or latent TB and compared with two distinct control groups consisting of BCG-vaccinated blood donors and hospitalized non-TB individuals.

**Results:**

A robust humoral response was detected in patients with active TB whereas antibodies against lipolytic enzymes were infrequently detected in either uninfected groups or in subjects with latent infection. High specifity levels, ranging from 93.9% to 97.5%, were obtained for all four antigens with sensitivity values ranging from 73.4% to 90.5%, with Rv3452 displaying the highest performances. Patients with active TB usually exhibited strong IgG responses but poor IgM responses.

**Conclusion:**

These results clearly indicate that the lipolytic enzymes tested are strongly immunogenic allowing to distinguish active from latent TB infections. They appear as potent biomarkers providing high sensitivity and specificity levels for the immunodiagnosis of active TB.

## Introduction

Tuberculosis (TB) which is caused by *Mycobacterium tuberculosis* (*Mtb*), remains one of the leading causes of death in the world [1]. Approximately one-third of world population is currently infected with *Mtb*. According to the WHO, around 9.2 million new TB cases and 1.7 million deaths occur every year [2]. The increasing global health burden of TB is further aggravated by the alarming increase of the number of people living with HIV and the emergence of multi- and extensively drug-resistant *Mtb* strains, which requires a longer, more costly therapeutic regimen [3], [4]. One of the best prognoses for TB comes with early diagnosis of the infection and immediate implementation of appropriate chemotherapy. In many countries, especially in resource constrain areas, the diagnosis of TB largely relies on the detection of acid-fast bacilli in sputum in conjunction with assessment of clinical symptoms and X-ray radiographic evidence. However, these evaluations offer suboptimal diagnosis performances and are time-consuming. Currently, the tuberculin skin test based on the use of purified protein derivative (PPD) is the only available immune-based diagnostic test for clinical use in most developing countries. However, prior vaccination with BCG and cross-reaction with other mycobacterial species result in a poor specificity of this century-old test [5]. In addition, this test does not permit to clearly distinguish between the active and latent form of TB infection. Thus, the clinical relevance of PPD-based skin test appears not highly reliable [6].

In recent years, important efforts have been made to develop and rapid TB diagnosis tests. Tests based on antigens suffer from poor sensitivity and specificity to diagnose TB cases with smear-negative sputum samples [7], [8]. Immunoassays based on the detection of antibodies against *Mtb* antigens appear as an alternative to the TB diagnosis especially in low-resource countries [9]. In this context, numerous antigens able to trigger specific antibody responses in TB patients have been identified and characterized, albeit no single antigen appears to be ideal yet for the development of immunodiagnosis tests [8], [10], [11]. Therefore, identification of appropriate *Mtb* antigens suitable for immunodiagnosis, that offers ease of detection, high specificity and sensitivity allowing distinguishing patients with active disease from BCG-vaccinated and latently infected individuals are highly desirable. One of the potential strategies in developing new TB diagnostic assays consists in the identification of new candidate antigens, such as lipolytic enzymes, that rely on particular aspects of the physiology of the tubercle bacilli.

During infection, *Mtb* accumulates intracellular lipid-loaded inclusion bodies [12] whose lipids probably originate from the host cell membrane degradation [13], [14], [15], [16]. There is now strong evidence supporting that fatty acids also represent a source of carbon during dormancy [17], [18], [19]. *Mtb* stores fatty acids in the form of triacylglycerol (TAG) as it enters in the non-replicating persistence stage. Moreover, granulomas have been found to contain foamy macrophages that are cells bearing large amounts of neutral lipids surrounded by phospholipids in their cytoplasm. These lipid bodies are induced by the internalisation of bacteria and therefore providing a carbon source for the pathogen [20]. Overall, these findings support the view that enzymes involved in lipid degradation may fulfill important physiological functions and may participate in the extraordinary capacity of survival of *Mtb* within the infected host. Acquisition of host lipids by *Mtb* is likely to be performed by lipolytic enzymes, such lipases and phospholipases, including the cutinase family enzymes [14]. To date, except Rv0183 identified as a monoglyceride lipase [21], lipases belong to the *Lip* family which includes 24 lipid/ester hydrolases called LipC to LipZ based on their presence of the consensus sequence GXSXG characteristic of members of the α/β hydrolase fold family. Due to its location in the cell wall, Rv0183 has been proposed to participate in bacterial cell interactions [22]. Other lipases have been classified as cutinases, which are serine esterases active on a large panel of substrates such as cutin from plants [23], TAGs [24] and phospholipids [25]. *Mtb* possesses at least seven genes related to the cutinase family. Among this family, two enzymes are secreted, Rv1984c which preferentially hydrolyses medium-chain carboxylic esters and monoacylglycerols and Rv3452 which behaves like a phospholipase A2 and is able to induce macrophage lysis [16]. From their central role in mycobacterial metabolism and their participation as interacting players between bacilli and host cells, it may be inferred that specific antibodies against lipolytic enzymes may be induced at different stages of the infection process, therefore providing potential relevant diagnosis markers of TB. This hypothesis has recently been examined with respect to LipY (Rv3097c, PE30), a lipase that is a member of the PE family. LipY expresses TAG hydrolase activity [26]. It appears strongly associated to the mycobacterial cell surface [27] and its accessibility to the host immune system was supported by the induction of a strong specific humoral response in TB patients living in an endemic area [27]. This prompted us to extend these initial observations and to investigate whether other *Mtb* lipolytic enzymes also induce specific immune responses in patients with either active or latent infections.

Herein, we have examined the humoral response against four distinct *Mtb* lipolytic enzymes in different patient groups. These antigens including LipY, the monoacylglycerol lipase Rv0183 as well as two cutinase-like proteins (Rv1984c and Rv3452) were chosen as members of different lipolytic enzyme families characterized by distinct expression profiles and substrate specificities.

## Results

### Expression and purification of *M. tuberculosis* lipolytic enzymes

The main proteomic and enzymatic characteristics of the Rv0183, Rv1984c, Rv3097c and Rv3452 are depicted in Table 1. These data indicate that all four enzymes can be distinguished with respect to the presence of particular protein domains, enzymatic activity, localization and genetic distribution. Briefly, whereas Rv0183 could be excreted from the cell wall [21], Rv1984c and Rv3452 have been reported to be secreted [16] and Rv3097c (LipY) has been shown to be strongly associated to the mycobacterial cell wall and surface-exposed [27]. This latter is also the only one that possesses a N-terminal PE domain which has been suggested to participate in the regulation of the catalytic activity of the lipase [27]. In addition, distribution of *lipY* is only restricted to some mycobacterial species, including members of the *Mtb* complex as well as *M. marinum,* in which the PE domain is substituted by a PPE domain [27]. Rv0183 is found in most mycobacterial species [21]. Cutinases have been found in phytopathogenic bacteria and in various environmental mycobacterial species, such as *Mycobacterium vanbaalenii* and *Mycobacterium* sp. KMS, isolated from soil. In *Mtb*, Rv1984c (Cfp21) belongs to the region of differentiation 2 (RD2), which is deleted from BCG substrains derived from the original BCG Pasteur strain during year 1926-1931 [28]. Therefore, RD2 constitutes a potential source of specific antigens for TB immunodiagnosis [29]. Rv0183 and LipY have been shown to exhibit monoacylglycerol (MAG) and triacylglycerol (TAG) hydrolytic activities, respectively, whereas the two cutinase-like enzymes have been demonstrated to hydrolyse either medium-chain carboxylic esters and MAGs (for Cfp21) or phospholipids (for Rv3452).

**Table 1:** Proteomic and enzymatic characteristics of the four lipolytic enzymes.

Rv number	Name	Signal Peptide	Length (AA)	M (kDa)	pI	Secreted	RD region	Enzymatic activity	References
Rv0183	-	+	279	30.3	6.17	+	−	MAG hydrolase	[21], [22]
Rv1984c	Cfp21/Culp1	+	217	21.8	5.88	+	RD2	Lipase	[16], [54]
Rv3097c	LipY/PE30	−	437	45.0	4.26	ND	−	Lipase	[26], [27]
Rv3452	Cut4/Culp4	+	226	23.1	9.33	+	−	Phospholipase	[16], [54]

ND, not determined; M, molecular weight, pI, isoelectric point; RD, region of differentiation; MAG, monoacylglycerol.

All four enzymes were cloned and expressed as N-terminally His tagged recombinant proteins, either in *E. coli* for Rv0183, Rv1984c and Rv3452 or in *M. smegmatis* for LipY. Following overexpression, Ni^2+^-charged immobilized metal affinity chromatography was utilized to purify the proteins, subsequently analyzed by SDS-PAGE (Figure S1). Because LipY was found to be strongly associated to the mycobacterial cell wall, a method using N-lauroyl-sarcosine was developed to extract and purify this protein. This allowed us to obtain high yields of active LipY produced in a mycobacterial membrane environment and at high purity levels (Figure S1).

### IgG antibody response against LipY, Rv0183, Rv1984c and Rv3452 in active TB patients

All four proteins have been demonstrated to be either surface-exposed or secreted into the culture filtrate of *Mtb*, leading to the hypothesis that they are accessible to the immune system of the infected host [27]. However, little is known regarding their immunogenic properties. We, therefore, evaluated their role as antigens in clinical settings. Four individual indirect ELISA were optimized and used to analyse sera from distinct patient groups. First, the IgG response against the different selected antigens was determined in samples from polish (n = 62) and french (n = 43) patients with active TB or in subjects without active TB. Characteristics of the included subjects are provided in Table 2.

**Table 2:** Clinical characteristics of the populations.

		Number of patients			
Serum origin	Tuberculosis type	Total number of sera	Smear positive	Smear negative Culture positive	ELISPOT positive
	**Total TB patients**	105	82	23	N.A.
**Poland**	Pulmonary TB	62	56	6	N.A.
	Extra-pulmonary TB	0	0	0	N.A.
**France**	Pulmonary TB	37	24*	13	N.A.
	Extra-pulmonary TB	6	2	4	N.A.
	**Total LTBI patients**	49	0	0	49

*includes 2 HIV^+^ patients; TB, *tuberculosis* patients; LTBI, latent *tuberculosis* infection; N.A., non available.

**Figure 1:**
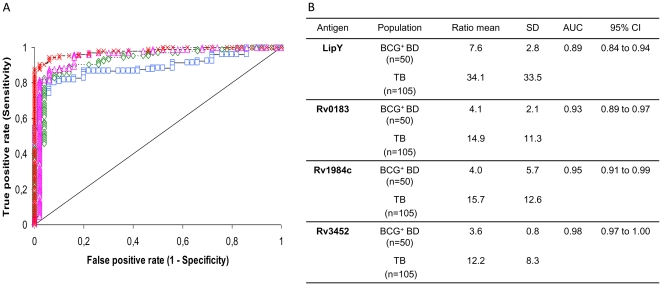
ROC analysis of the antibody response against four *M. tuberculosis* lipolytic enzymes. (**A**) Comparison of the ROC curves for IgG detection specific to LipY (blue), Rv0183 (pink), Rv1984c (green) and Rv3452 (red). The grey line represents the no-discrimination line from the left bottom to the top right corners. (**B**) Ratio mean, standard deviation (SD) and area under the ROC curve (AUC) derived from (A). The *p* value for active TB patients versus healthy controls (BCG^+^ BD) is <0.0001. BCG^+^ BD, BCG vaccinated blood donors; n, number of sera; CI, confidence interval; SD, standard deviation.

The receiver operating characteristic (ROC) curves, which describe the relationship between the sensitivity and specificity at any cut-off values, are depicted in Figure 1A. The ratio mean and the area under the curve (AUC) of each antibody response are provided in Figure 1B. The AUC for LipY, Rv0183, Rv1984c and Rv3452 were 0.89 (0.84–0.94), 0.93 (0.89–0.97), 0.95 (0.91–0.99) and 0.98 (0.97–1.00), respectively. Based on the AUC values, cut-off levels for each antigen were set to generate the optimal combination of specificity (96%) and sensitivity. AUC values were found to be significantly different between Rv3452 and LipY (*p*<0.0001), Rv3452 and Rv0183 (*p*<0.01) and Rv1984c and LipY (*P*<0.05).

**Figure 2:**
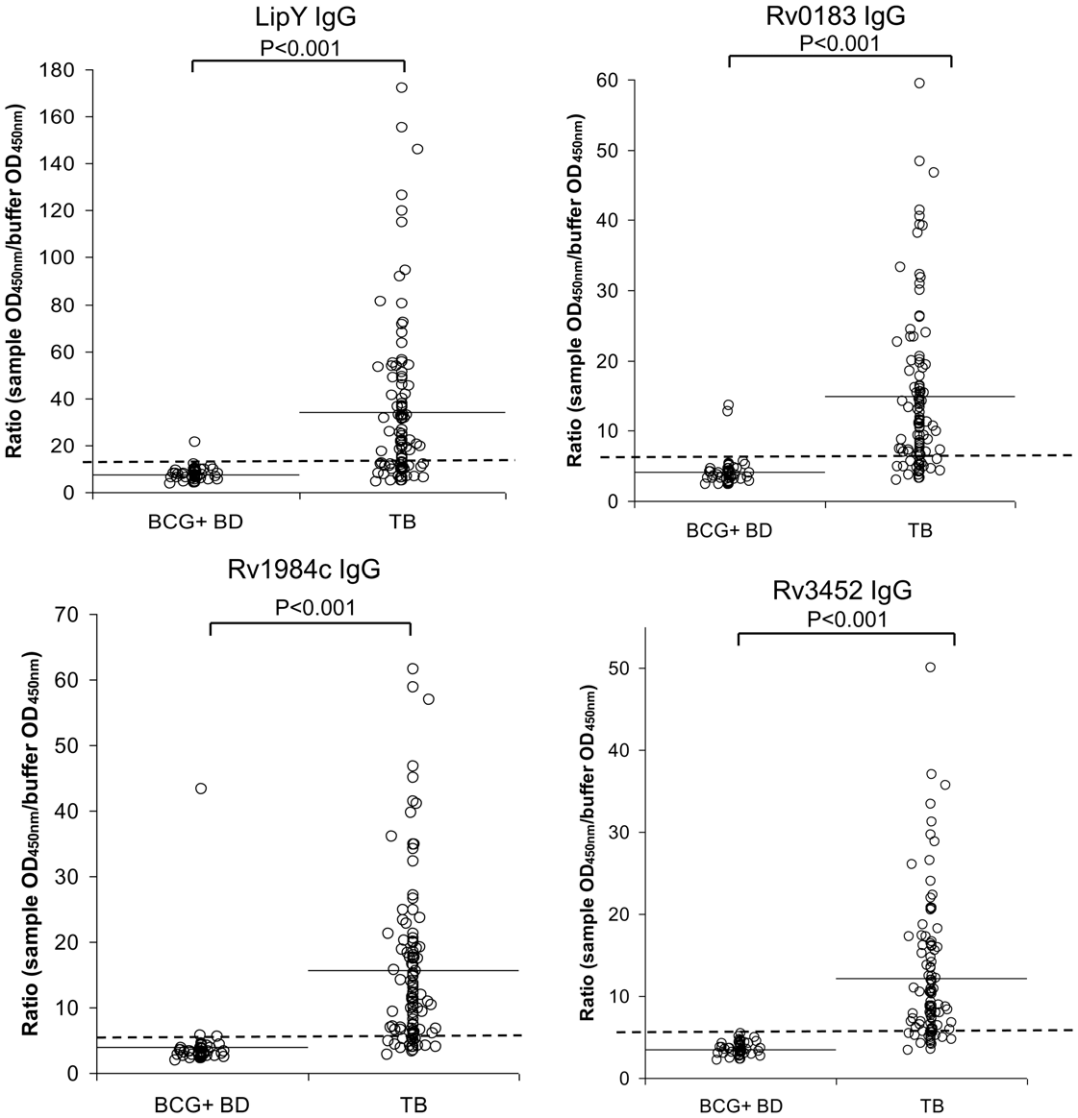
Comparison of the antibody IgG response to recombinant lipolytic enzymes in TB patients and healthy individuals. (**A**) LipY, (**B**) Rv0183, (**C**) Rv1984c and (**D**) Rv3452. Horizontal lines indicate the mean value, whereas dotted lines indicate the cut-off values, derived from the ROC curves. BCG^+^ BD, BCG vaccinated blood donors (n = 50), TB, active TB patients (n = 105).

The humoral IgG response to each antigen in active TB patients as opposed to BCG^+^ healthy controls is shown in Figure 2. The specificity to all four antigens was determined using three different populations: french BCG-vaccinated blood donor (BCG^+^ BD, n = 50), french hospitalized patients which were BCG-vaccinated, had no declared TB neither latent TB based on a negative IFN-ã release assay (IGRA) (TB^-^HP; n = 50) and french latent TB infection patients (LTBI; n = 49). As shown in Table 3, a specificity of 96% for all four antigens was achieved in the BCG^+^ blood donors. Very high specificity values were also obtained against the TB^-^HP population, ranging from 94% (for Rv3452) to 100% (for LipY). For the latently infected population, the specificity varied between 87.8% (for Rv1984c) to 95.9% (for LipY).

**Table 3:** Relative specificity and sensitivity of the IgG detection against *M. tuberculosis* antigens.

Subjects group (n)	LipY	Rv0183	Rv1984c	Rv3452
	**Specificity (%) (95% CI)**			
**Total control (149)**	97.3 (93.3 to 99.3)	93.3 (88.0 to 96.7)	94.0 (88.8 to 97.2)	94.6 (89.7 to 97.7)
French BCG^+^ BD (50)	96.0 (86.3 to 99.5)	96.0 (86.3 to 99.5)	96.0 (86.3 to 99.5)	96.0 (86.3 to 99.5)
French TB^-^ HP (50)	100.0 (92.9 to 100.0)	94.0 (83.5 to 98.7)	98.0 (89.3 to 99.9)	94.0 (83.5 to 98.7)
French LTBI (49)	95.9 (86.0 to 99.5)	89.8 (77.7 to 96.6)	87.8 (75.2 to 95.4)	93.9 (83.1 to 98.7)
	**Sensitivity (%) (95% CI)**			
**Total TB (105)**	73.4 (63.8 to 81.5)	81.0 (72.9 to 88.3)	81.9 (73.2 to 88.7)	90.5 (83.2 to 95.3)
Polish TB (62)	91.9 (82.2 to 97.3)	100.0 (94.6 to 100.0)	98.4 (91.7 to 100.0)	96.8 (88.8 to 99.6)
French TB (43)	46.5 (31.2 to 62.3)	53.5 (37.7 to 68.8)	58.1 (42.1 to 73.0)	81.4 (66.6 to 91.6)

n: number of subjects; BCG^+^ BD: BCG vaccinated blood donors, TB^-^HP: non-tuberculosis hospitalized patients, TB: tuberculosis patients, LTBI: latent tuberculosis infection.

As shown in Table 3, the sensitivity varied from 73.4% to 90.5%, Rv3452 being the more potent antigen when assessing the total TB population comprising both the polish and french patients. However, when both populations were analyzed separately, one could clearly distinguish an important sensitivity difference. Accordingly, the sensitivity of all four antigens was significantly higher in the polish TB population, ranging from 91.9% (82.2%–97.3%) for LipY to 100% (94.6%–100%) for Rv0183 compared to the french TB population, ranging from 46.5% (31.2%–62.3%) for LipY to 81.4% (66.6%–91.6%) for Rv3452 (*p*<0.05).

Overall these results clearly highlight the potential of lipolytic enzymes and the excellent performances of Rv3452 in the diagnosis of active TB.

### Serological responses in latent TB patients

T-cell responses, even for the most immunodominant antigens, including ESAT-6 and CFP-10, do not allow to discriminate between active TB infection and latent infection [30]. Likewise, ESAT-6 and CFP-10 antibodies are present in active TB patients but also in latent infection individuals, particularly in areas with high levels exposure to *Mtb* [31]. Several recent studies also highlight the insufficient specificity of serodiagnostic tests designed to detect active TB and used in TB epidemic regions [32], [33]. Therefore, we evaluated the specific IgG responses against all four antigens in sera from latent TB infected individuals proved by a positive result with IGRA. Positive rates for LipY, Rv0183, Rv1984c and Rv3452 were 4.1%, 10.2%, 12.2% and 6.1%, thus highlighting the very low response elicited against the four antigens tested in latently infected patients.

Therefore, the difference between active TB and latent TB groups in humoral responses to all four lipolytic enzymes suggests that these antigens could serve to successfully discriminate active from latent TB infections.

### Comparison of the serological responses with respect to the microbiological status

Positive IgG sera were next compared with the bacteriological status determined by microscopy and culture (Table 4). The data indicate the presence of a different response according to the result of microscopic examination for the presence of acid-fast bacilli (AFB). For instance, anti-LipY antibodies were found in 82.9% in smear-positive patients and in 64.7% in smear-negative culture-positive patients. The proportion of patients suffering from active TB without AFB detected by microscopic examination was found to be more pronounced in the french than in the polish population. In this analysis, Rv0183 appeared as the least potent marker as specific antibodies were detected in only in 52.2% of smear-negative patients. In contrast, regarding Rv3452, antibodies were detected in 92.7% of the smear-positive patients and 87.0% in smear-negative patients (Table 4).

**Table 4:** Number of IgG positive response of TB serum according to bacteriological status.

	Number of TB sera (% positive sera)		
Antigen	Serum origin (n = 105)	Smear positive (n = 82)	Smear negative Culture positive (n = 23)
**LipY**	Poland	53 (94.6)	6 (100.0)
	France	15 (57.7)	5 (29.4)
	**Total**	**68 (82.9)**	**11 (64.7)**
**Rv0183**	Poland	56 (100.0)	6 (100.0)
	France	17 (65.4)	6 (35.3)
	**Total**	**73 (89.0)**	**12 (52.2)**
**Rv1984c**	Poland	55 (98.2)	6 (100.0)
	France	18 (69.2)	7 (41.2)
	**Total**	**73 (89.0)**	**13 (76.5)**
**Rv3452**	Poland	54 (96.4)	6 (100.0)
	France	22 (84.6)	14 (82.4)
	**Total**	**76 (92.7)**	**20 (87.0)**

n, number of sera; N.A., non availaible; n total of polish sera  =  62; n total of french sera  =  43; n of smear positive of polish sera  =  56; n of smear positive in french sera  =  26; n of smear negative and culture positive of polish sera  =  6; n of smear negative and culture positive of french sera  =  17.

## Discussion

Antibody responses are typically investigated in infectious diseases where antibody production strongly affects pathogenesis and the outcome of the infection. Antibodies are also produced in infections caused by intracellular pathogens, including TB, where the protective immunity is primarily elicited by the T-cell response [34]. Although approximately 90% of TB patients produce antibodies to *Mtb* proteins [35], much remains to be learnt with respect to the correlation between antibody production, antibody specificity and disease progression. During the last few years, an important number of studies have been dedicated on circulating antibodies in TB patients with the goal of evaluating them as biomarkers of active disease. However, despite of intense research in this particular field, the World Health Organization has, for the first time, delivered negative recommendations against the use of the current commercial TB immunoassays based on antibodies detection because they may be doing more harm than good in high-burden countries. Low sensitivity means increased false negative results, which increase mortality and ongoing transmission of TB. Low specificity means more false positive results and then, patients might be given 6 months of potentially toxic treatment [36]. However, in the same time the WHO has claimed that biomarker discovery remains a major challenge to the establishment of an efficient serodiagnostic test for the tuberculosis (http://www.stoptb.org/assets/documents/global/plan/TB_GlobalPlanToStopTB2011-2015.pdf).

In this context, we reasoned that efficient biomarkers for the diagnosis of TB should derive from novel concepts with respect to the biology/physiology of *Mtb*. In our recent studies, several lipolytic enzymes of *Mtb* were characterized both biochemically and enzymatically [16], [21], [22], [27], allowing us to propose that these enzymes, or at least some of them, are very likely to participate in lipid metabolism during dormancy and/or during reactivation [14]. This view is supported by a recent study conducted by Low *et al.* demonstrating extensive accumulation and degradation of TAGs in the bacilli during entry into and exit from hypoxia-induced dormancy, respectively [37]. Moreover, these processes are accompanied by dynamic appearance and disappearance of intracellular TAG lipid particles [19]. Importantly, reduction of TAG levels coincides with an increase in cellular TAG lipase activity in regrowing bacilli, leading to the conclusion that TAG utilization is essential for the regrowth of mycobacteria during their exit from the non-replicating state [37]. Therefore, one may hypothesize that lipolytic enzymes such as LipY, which is not expressed under normal growth conditions [27], are mainly induced and expressed under reactivation conditions, and hence, may represent useful biomarkers to detect reactivated forms of TB.

Herein, we provide for the first time evidence that proteins Rv0183 and Rv3452 induce robust humoral responses in TB patients. Regarding Rv1984c, Wang *et al*. reported an anti-Rv1984c IgG response in pulmonary TB patients with a sensitivity of 46.2% and a specificity of 100% [38]. In our study, a higher sensitivity level was achieved (81.9%). This difference may be attributed to the purity rate of the recombinant protein, the optimization of the ELISA and/or differences in the populations studied.

With respect to LipY, these data are in agreement with those of Mishra *et al*. [27] reporting a humoral response against LipY in patients suffering from pulmonary TB or extrapulmonary TB. However, among the four antigens, LipY appears like the least attractive, albeit it was expressed and purified from *M. smegmatis,* which allows more accurate processing of gene products, including folding and eventual post-traductional modifications, than expression in *E. coli* [39], [40]. In contrast to Rv1984c and Rv3452, which have proposed be secreted by *Mtb*, LipY has been shown to be strongly associated to the mycobacterial cell wall and surface-exposed. These differences in localization may influence the intensity of the immune response, although this remains to be further investigated. Among the studied antigens, Rv3452 exhibits the best performances to diagnose active TB. Several studies have demonstrated that the detection of antibodies directed against multiple antigens provides an improvement in sensitivity compared to the use of a single antigen [41], [42]. However, as shown in Table S1, the sensitivity in total TB patients was not significantly enhanced (93.3% when individually combined together with each single antigen versus 90.5% when assayed alone (Table 3)). Moreover, this little gain in sensitivity was counter-balanced by a decrease in specificity value (85.2% versus 94.6% when assayed alone).

Poland and France are low TB endemic areas. The prevalence of the TB in Poland is of 32 cases per 100,000 inhabitants, whereas in France, the prevalence is of 7.3 cases to 100 000 inhabitants [2]. However, our data highlight an important difference with respect to the IgG response against all fours antigens between polish and french TB patients, that is reflected by a significant difference in the sensitivity between the two populations (91.9% [82.2%–97.3%] versus 46.5% [31.2%–62.3%] for LipY or 100.0% [94.6%–100.0%] versus 53.5% [37.7%–68.8%] for Rv0183). This discrepancy may be linked to the microbiological status of the patients analyzed. Previous studies have shown that detection of antibodies against the 38-kDa protein or lipoarabinomannan has high sensitivity and specificity in active TB patients, but is poor in detecting a reponse in smear-negative TB patients [43], [44]. Indeed, in our study, only 9.7% of the patients in the polish population is smear-negative (6 out of 62), compared to 39.5% (17 out of 43) in the french population (Table 2). Alternatively, the difference in sensitivity may be influenced by disease progression and by the extent of cavitation which affects the antibody response, as suggested in previous studies [45], [46], [47]. Indeed, the presence or absence of cavitary lesions has a significant, reproducible effect on the profile of antigens that are recognized by the antibodies [46]. It had been proposed that the presence of antibodies to some antigens in cavitary TB patients suggests that *in vivo* either these antigens are expressed primarily during extracellular replication of the bacilli in liquified caseous material or that these antigens are accessible to the immune system only during this particular stage of the disease. It remains, however, to be established whether the observed differences between french and polish populations correlate with differences in their radiological status even when they are bacteriologically similar.

Another possible explanation of the difference in sensitivity between french and polish populations may also rely on exposition to various environmental mycobacteria, as this may also impact in generating cross-reactive antibodies, especially if antigens are not *Mtb*-specific. However, further studies are required to evaluate the eventual impact/contribution of infection with environmental mycobacteria on the accuracy of these antigens.

In addition, among the french patients, 6 suffered from extra-pulmonary TB cases and two were HIV co-infected, all these cases being absent from the polish population. Only two extra-pulmonary TB cases contained IgG against LipY, Rv0183 and Rv1984c. In contrast, 5 out of 6 of these patients presented an anti-Rv3452 IgG reponse (data not shown). Therefore, it appears very likely that the lower sensitivity values obtained with the different markers in the french population is directly associated to the occurrence of a highly heterogenous population with respect to the bacteriological and clinical status. It is, however, noteworthy that some of the markers studied, particularly Rv3452, exhibited very good serodiagnostic properties in both population (93.9% to 96% of specificity in the control populations, 96.8% of sensitivity in the polish population and 81.4% in the french population), thus suggesting that this marker is of high value for future diagnostic developments. This is also strengthened by the fact that Rv3452 IgG antibodies were detected in extra-pulmonary TB cases and in both HIV/TB co-infected patients (data not shown).

We have also investigated whether the active TB patients were able to mount specific IgM antibody responses against the four antigens. In contrast to IgG, very few patients exhibited specific IgM responses to the various antigens (Table S2). For instance, only 9.5% patients displayed IgM seropositivity to Rv3452 whereas 90.5% were IgG-positive. Comparing both IgG and IgM responses indicated that some patients displayed specific IgM, but not IgG, antibodies. Indeed, two patients developed anti-LipY IgM antibodies but not anti-LipY IgG antibodies, and sera from four patients contained anti-Rv0183 IgM but not IgG antibodies (Table S2). IgM antibodies are usually the first to appear in response to any antigen and are expected to be produced in larger quantities following primary TB infection and to decline in more advanced phases. Several authors suggested that IgM are produced mainly during the early phase of primary TB infection [48]. One may therefore hypothesize that the IgM-positive patients were diagnosed at an early stage of the infection process.

Identification of new targets is also required for the detection of LTBI. In our study, no discrimination was observed between control individuals and latent TB infected people (*p*>0.05). IGRA represent the methods generally used to diagnose LTBI. For these purposes, several antigens have been successfully used, like ESAT-6 and CFP-10 in QuantiFERON® and T-SPOT-TB® tests. Interestingly, Rv1984c has also previously been shown to induce IFN-ã release in the Guinea pigs, mice and TB patients [49], [50], [51]. Therefore, evaluating the release of IFN-ã after stimulation of T cells with LipY, Rv0183 or Rv3452 may represent an alternative to IGRA option, particularly if it permits to distinguish latent from active TB.

In conclusion, to replace the gold standard culture, an immunoassays should possess sensitivities of over 80% and test specificities of over 95% according to the recommendations of the WHO [2]. Using the *Mtb* lipolytic enzymes tested in the present study these criteria were achieved. Further studies, including field trials, are now required to evaluate the potential and usefulness of Rv3452 for TB diagnosis in smear-negative and HIV-infected persons in countries highly afflicted by HIV and TB.

## Materials and Methods

### Collection of blood serum

Fifty sera of healthy blood donors from France vaccinated with BCG (negative for HIV) and 50 hospitalized non-TB subjects vaccinated with BCG and negative for TB as detected by IGRA tests (T-SPOT-TB™, Oxford Immunotec, UK) were analyzed in this study. In addition, two groups of patients with active TB were included. In the first group, sera from 62 patients suffering from active pulmonary TB were collected in Poland. As detailed in Table 2, among these patients 56 patients were positive by smear microscopy. The second group comprises 43 french patients with active TB (37 patients with pulmonary TB and 6 extrapulmonary TB, with 26 smear-positive patients, including 2 HIV-positive patients. In another group of 49 subjects, latent TB infection was diagnosed using the T-SPOT-TB™ (Table 2).

### Ethics statement

The sera of healthy blood donors were supplied by the Center of Blood Transfusion (Centre de transfusion sanguine de Lille, France). Their use is in accordance with all relevant national and European Union regulations. Patients were included after having given written consent (AFSSAPS n° 2010-A00422-37), in compliance with the Helsinki declaration and after approval of the local ethics committee (Comité Consultatif de Protection des Personnes pour la Recherche Biomédicale de Nimes, France). All samples were treated anonymously. Antibodies were produced in New-Zealand rabbits using recombinant antigen following approval by Ms L. De-Coninck, the person duly entlitled at Bio-Rad to conduct animal experimentations in the respect of ethic rules, agreement N° 59-580140, delivered by the Prefecture du Nord, France. There was no specific ethic comity but all experimentions were done following standard protocols, examined and approved by Ms L. De-Coninck, who also warrants that all experimentations were performed with respect the required ethical rules. Articles R214-87 to R214-122 and article R215-10 of the French Rural Code in conjunction with the French administrative order dated 19 April 1988 establish eligibility conditions for authorization to conduct experiments on animals. Permission is given to a person to review the ethical compliance of protocol conducted in animals according to the recommendations of the French and European Community guidelines for laboratory handling, and to allow research to be carried out when these criteria are met.

### Bacterial strains

*Escherichia coli* Rosetta pLysS was used for the expression of recombinant Rv0183 and Rv1984c, whereas C41(DE3) was used to overproduce Rv3452 [16], [21]. LipY was expressed from recombinant *M. smegmatis* mc^2^155, as reported earlier [27].

### *M. tuberculosis* lipolytic enzymes expression constructs

The *Rv0183*, *Rv1984c*, *Rv3452* genes lacking their predicted signal peptide and *Rv3097c* were amplified from cosmids MTCI28.23, MTCY39, MTCY13E12 and BAC 48, respectively (obtained from the Pasteur Institute). *Rv0183*, *Rv1984c* and *Rv3452* were cloned into pDest14 (Invitrogen) using the gateway technology, giving rise to pDest14-His-*Rv0183*, pDest14-His-*Rv1984c* and pDest14-His-*Rv3452*, respectively [16], [21]. These constructs allow the expression of N-terminal His-tagged fusion proteins containing a TEV protease recognition site. *Rv3097c* was cloned into the acetamide-inducible vector pSD26 [52], as described previously [27].

### Expression and purification of Rv0183, Rv1984c and Rv3452

Recombinant proteins were expressed and purified as described previously [16], [21]. Briefly, *E. coli* strains carrying pDest14-His-*Rv0183,* pDest14-His-*Rv1984c* or pDest14-His-*Rv3452* were grown overnight in LB medium at 37°C and then diluted in Terrific Broth. Protein expression was induced with 1 mM isopropyl β-D-thiogalactoside (IPTG) and the temperature decreased to 25°C for another 16 hrs. Bacteria were lyzed in 50 mM Tris/HCl pH 8.0, 150 mM NaCl, 1 mM EDTA, 0.1% Triton X-100, 0.25 mg/ml lysozyme supplemented with 10 µg/ml DNAseI and 20 mM MgSO_4_. For purification of Rv0183, the supernatant was separated from the cell debris and loaded onto a Ni^2+^-Agarose column After washing with buffer A (10 mM Tris/HCl pH 8.5, 150 mM NaCl) containing 10 mM imidazole, the protein was eluted with buffer A supplemented with 500 mM Imidazole. Fractions were pooled and further purified by size exclusion chromatograph (Superdex 200) in buffer A.

Recombinant Rv1984c and Rv3452 were expressed only in an insoluble form [16]. After washing inclusion bodies were resuspended overnight at 4°C in 50 ml of buffer A containing 8 M urea. After centrifugation at 17,000 *g*, solubilized proteins were loaded onto a Ni^2+^-NTA resin equilibrated with buffer A containing 8 M urea. Proteins were eluted in the same buffer supplemented with 250 mM imidazole. Fractions were pooled and incubated with 10 mM DTT for 1 hr at 4°C. Proteins were diluted 20 times in the appropriate buffer selected from the refolding plates [53] and left to refold overnight at 4°C under gentle agitation. Soluble proteins were concentrated and further purified by size-exclusion chromatography column (Superdex 200) equilibrated with the appropriate buffer. Pure rRv1984c and rRv3452 were obtained in 10 mM Tris/HCl (pH 8) containing 300 mM NaCl and 50 mM 2-(N-cyclohexylamino)ethane sulfonic acid (pH 9) refolding buffers, respectively.

For all three proteins, N-terminal His tags were removed by TEV digestion and purified by exclusion from a Ni^2+^-NTA agarose beads. Protein concentration was calculated from the OD_280nm_ using the extinction molar coefficients 0.858, 0.78, 0.651 M^−1^.cm^−1^ for Rv0183, Rv1984 and Rv3452, respectively, and stored at −80°C until further use.

### Expression and purification of LipY

Expression of recombinant LipY was performed using *M. smegmatis* mc^2^155 strain as previously reported [27] with some modifications. Briefly, *M. smegmatis* carrying pSD26_LipY was used to inoculate 4 ml of 7H9 medium containing 50 µg/ml hygromycin B and used to inoculate 400 ml of culture medium for large-scale production. Cells were grown at 37°C with shaking (220 rpm) until an OD_600nm_ value of 3 was reached. Expression of recombinant proteins was induced for by adding 0.2% acetamide for another 16 hrs. Bacteria were harvested and resuspended in ice-cold buffer consisting of 10 mM Tris/HCl buffer (pH 8.0) containing 150 mM NaCl and 1% N-lauroyl-sarcosine (sarcosyl) and subsequently broken. The supernatant was recovered whereas the pellet was resuspended again and sonicated thrice during 30 s with 30 s breaks between each cycle and stirred overnight at 4°C. Both supernatants were then pooled and loaded onto a Ni^2+^-NTA resin equilibrated with 10 mM Tris-HCl buffer (pH 8.0) containing 150 mM NaCl and 1% sarcosyl. The column was subsequently washed with buffer without detergent prior elution with increasing concentrations of imidazole. Fractions containing pure LipY were pooled, dialysed overnight against 10 mM Tris-HCl buffer (pH 8.0) containing 150 mM NaCl and concentrated by ultrafiltration to a final concentration of 0.5 mg/ml.

### Production and purification of polyclonal antibodies

Anti-LipY antibodies were produced in two rabbits (New-Zealand) using recombinant LipY as antigen. One rabbit was injected subcutaneously and one rabbit was injected intramuscularly. Each rabbit was injected four times every two weeks with 250 µg of antigen mixed with an equal volume of incomplete Freund's adjuvant (Sigma). Polyclonal LipY antibodies were purified by affinity chromatography on Protein A agarose (Streamline rProtein A, Ge Healthcare). Specificity of the antibodies response was assessed by an ELISA. A similar protocol was carried out with Rv0183, Rv1984c and Rv3452. All experiments were performed in accordance with French and European Community guidelines for laboratory animal handling.

### Preparation of HRP-conjugates for ELISA

To prepare the HRP (horseradish peroxydase)-conjugates derived from each polyclonal antibody, Ab/HRP (Roche) labeling ratios of 1/4 (mol/mol) were used. HRP was oxidized with 0.1 M sodium periodate. Oxidation was blocked with 0.85 M ethylene glycol and oxidized HRP was purified on a PD-10 desalting column (GE Healthcare) with 0.1 M carbonate buffer. Fraction were monitored by measuring the OD_280nm_, pooled and used for the binding with antibodies. Coupling was performed in 1 M carbonate buffer overnight in the darkness and then stopped with 2.5% sodium borohydride for 15 min at room temperature in the darkness. The conjugate was then dialyzed against 0.01 M PBS overnight at 4°C. Protein concentration was determined using the BCA kit (Pierce) according to the manufacturer's instructions.

### Antibody detection by ELISA

For the detection of specific IgG antibodies, 100 µl of Individual recombinant proteins (1 µg/ml for Rv0183 and 3 µg/ml for Rv3452, 4 µg/ml for LipY and Rv1984c), were used to coat the wells in 96-well microtiter plates (Nunc) overnight. Then, the wells were blocked twice with 300 µl of blocking buffer (5% saccharose, 0.1% bovine serum albumin (BSA) in PBS), 100 µl of serum (diluted 1∶100) were added and incubated for 1h at 37°C. After washing, the bound antibodies were detected by 100 µl of HRP-conjugated mouse anti-human IgG (20 ng/ml) (Bio-Rad) incubated for 1 h at 37°C. After a last washing step, 200 µl of enzymatic substrate (tetramethylbenzidine [TMB] and peroxide hydrogen) was added and incubated at room temperature before stopping the reaction by 100 µl of 1 N sulfuric acid. Intensity of the reaction was assessed by measuring the OD_450nm_ in an ELISA plate reader (PR 3100, Bio-Rad).

For the detection of specific IgM antibodies, 100 µl of goat anti-IgM antibodies (2 µg/ml) (Bio-Rad) were coated in 96-well microtiter plates overnight at 4°C. After washing and incubation in blocking buffer, 100 µl of each serum (diluted 1∶100) were added and incubated for 1 h at 37°C. The various antigens (0.1 µg/ml for LipY and Rv1984c, 1 µg/ml for Rv0183 and Rv3452) were mixed with their corresponding rabbit IgG-HRP conjugates (1 µg/ml) prior to the addition of 100 µl of the mix in the wells. After incubation for 1 h at 37°C, plates were washed and detection was performed by adding 200 µl of TMB and peroxide hydrogen and stopped by adding 100 µl of sulfuric acid. The OD_450nm_ was measured using an ELISA plate.

### Data management and statistical analysis

All data were entered into a Microsoft Office Excel file. The ratio value corresponding to the sample OD_450nm_/buffer OD_450nm_ was determined for each sample. The mean and standard deviation of the ratio of individual groups for antibody/antigen responses to each marker were calculated. The receiver operating characteristic (ROC) curves of the ratio values for antibody responses to each marker were plotted using Analyse-it software and the area under the curves and 95% confidence intervals (95% CI) for responses to each antigen were calculated. Individuals were scored as positive for the specific antibody response when their ratio value was greater than or equal to the cut-off value. Results of each group of patients were compared using the Student *t* test and X^2^ test.

## Supporting Information

Figure S1 **SDS PAGE analysis of the purified lipolytic enzymes used in this study.** MW: Molecular weights are presented in the left margin. Rv0183 and LipY were loaded onto a 12% polyacrylamide gel while Rv1984c and R3452 were loaded onto a 15% polyacrylamide gel. Rv0183 (5 µg), LipY (4 µg), Rv1984c (5 µg) and Rv3452 (10 µg).
(PDF)

Table S1 **Relative specificity and sensitivity of the IgG detection against combination of** ***M. tuberculosis*** **antigens.**
(PDF)

Table S2 **IgM and IgG reactivity against** ***M. tuberculosis*** **antigens alone or in combination in the active TB population.**
(PDF)
