# Supplementary material for: Mycobacterium tuberculosis Lipolytic Enzymes as Potential Biomarkers for the Diagnosis of Active Tuberculosis
Source: PLoS One. 2011 Sep 22;6(9):e25078. doi: 10.1371/journal.pone.0025078 (PMC3178603; doi:10.1371/journal.pone.0025078)
Supplement: Figure S1 — SDS PAGE analysis of the purified lipolytic enzymes used in this study. MW: Molecular weights are presented in the left margin. Rv0183 and LipY were loaded onto a 12% polyacrylamide gel while Rv1984c and R3452 were loaded onto a 15% polyacrylamide gel. Rv0183 (5 µg), LipY (4 µg), Rv1984c (5 µg) and Rv3452 (10 µg). (PDF) [file pone.0025078.s001.pdf]

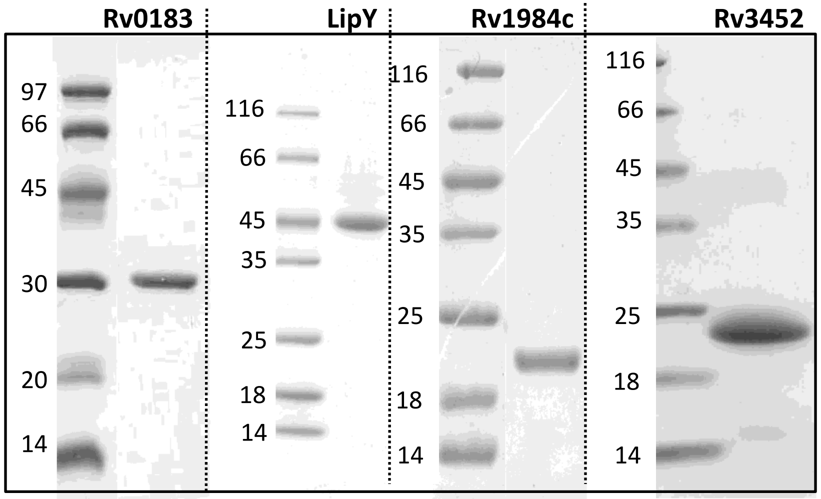

Figure S1. SDS PAGE analysis of the purified lipolytic enzymes used in this study. MW: Molecular weights are presented in the left margin. Rv0183 and LipY were loaded onto a 12% polyacrylamide gel while Rv1984c and R3452 were loaded onto a 15% polyacrylamide gel. Rv0183 (5 µg), LipY (4 µg), Rv1984c (5 µg) and Rv3452 (10 µg).
