# Supplementary material for: Mycobacterium tuberculosis Lipolytic Enzymes as Potential Biomarkers for the Diagnosis of Active Tuberculosis
Source: PLoS One. 2011 Sep 22;6(9):e25078. doi: 10.1371/journal.pone.0025078 (PMC3178603; doi:10.1371/journal.pone.0025078)
Supplement: Table S1 — Relative specificity and sensitivity of the IgG detection against combination of M. tuberculosis antigens. (PDF) [file pone.0025078.s002.pdf]

Table S1. Relative specificity and sensitivity of the IgG detection against combination of *M. tuberculosis* antigens.

Subjects group	Rv3452 and LipY	Rv3452 and Rv0183	Rv3452 and Rv1984c	All four antigens
		Specificity (%) (95% CI)		
**Total control (149)**	91.9 (92.0 to 95.8)	90.6 (84.7 to 94.8)	91.3 (85.5 to 95.3)	85.2 (78.5 to 90.5)
BCG^{+} BD (50)	92.0 (80.8 to 97.8)	92.0 (80.8 to 97.8)	94.0 (83.5 to 98.7)	86.0 (73.3 to 94.2)
TB^{-}BD (50)	94.0 (83.5 to 98.7)	92.0 (80.8 to 97.8)	92.0 (80.8 to 97.8)	92.0 (80.8 to 97.8)
LTBI (49)	89.8 (77.8 to 96.6)	87.8 (75.2 to 95.4)	87.8 (75.2 to 95.4)	71.4 (56.7 to 83.4)
		Sensitivity (%) (95% CI)		
**Total TB (105)**	92.4 (85.5 to 96.7)	92.7 (86.1 to 96.8)	92.7 (86.1 to 96.8)	93.3 (86.8 to 97.3)
Polish TB (62)	98.4 (91.3 to 100.00)	100.0 (96.7 to 100.0)	100.0 (96.7 to 100.0)	100.0 (96.7 to 100.0)
French TB (43)	83.7 (69.3 to 93.2)	81.4 (66.6 to 91.6)	81.4 (66.6 to 91.6)	83.7 (69.3 to 93.2)

n: number of subjects; BCG^{+} BD: BCG vaccinated blood donors, TB^{-} HP: non-tuberculosis hospitalized patients, TB: *tuberculosis* patients, LTBI: latent *tuberculosis* infection
