# Supplementary material for: Mycobacterium tuberculosis Lipolytic Enzymes as Potential Biomarkers for the Diagnosis of Active Tuberculosis
Source: PLoS One. 2011 Sep 22;6(9):e25078. doi: 10.1371/journal.pone.0025078 (PMC3178603; doi:10.1371/journal.pone.0025078)
Supplement: Table S2 — IgM and IgG reactivity against M. tuberculosis antigens alone or in combination in the active TB population. (PDF) [file pone.0025078.s003.pdf]

Table S2. IgM and IgG reactivity against *M. tuberculosis* antigens alone or in combination in the active TB population.

	Number of positive patients (n=105)				
	**LipY**	**Rv0183**	**Rv1984c**	**Rv3452**	**All four antigens**
IgM	7	11	9	10	11
IgG	77	85	86	95	98
IgM + IgG	5	7	7	10	10
